# Herpesviruses and Autophagy: Catch Me If You Can!

**DOI:** 10.3390/v2010314

**Published:** 2010-01-26

**Authors:** Yolaine Cavignac, Audrey Esclatine

**Affiliations:** INSERM U756 and Université Paris-Sud 11, Faculté de Pharmacie, 5 rue Jean-Baptiste Clément 92290 Châtenay-Malabry, France; E-Mail: yolaine.cavignac@u-psud.fr

**Keywords:** autophagy, Herpesvirus, HSV-1, cytomegalovirus, HHV-8, immunity, autophagosome

## Abstract

Autophagy is an evolutionarily conserved cellular degradation pathway involving the digestion of intracellular components via the lysosomal pathway. The autophagic pathway constitutively maintains cellular homeostasis by recycling cytoplasmic organelles and proteins, but it is also stimulated by environmental stress conditions, such as starvation, oxidative stress, and the accumulation of misfolded proteins. It also acts as a cellular defense mechanism against microorganisms by contributing to both the innate and adaptive immunity, and by eliminating intracellular pathogens (xenophagy). There is growing evidence that host cells try to control Herpesvirus infections by activating the autophagic machinery. However, it is well-known that Herpesviruses are smart pathogens and several, such as HSV-1, HCMV and HHV-8, are known to have developed numerous defense strategies for evading the host’s immune response. Inhibition of the antiviral autophagic mechanism has also been reported. Autophagy has also been shown to enhance the major histocompatibility complex presentation of at least two viral proteins, the EBV-encoded EBNA-1 and the HSV-1 encoded gB. In this review, we present an overview of recent advances in our understanding of the complex interplay between autophagy and Herpesviruses.

## 1. Overview of Autophagy

Macroautophagy (referred to as “autophagy” below) involves the formation of a double membrane-bound vacuole, known as an autophagosome, which ultimately fuses with the lysosomal compartment to degrade the sequestered material [1]. Under normal conditions, basal autophagy has a housekeeping function, allowing cells to recycle organelles and long-lived proteins, but autophagy is also induced in response to various forms of stress, such as starvation, depletion of growth factors, low energy levels, hypoxia, oxidative stress, ER stress and pathogen infection. Under these conditions, the upregulation of autophagy enables the cell to survive by providing amino acids and energy, by clearing damaged proteins and organelles, and by eliminating intracellular pathogenic microbes via a process known as xenophagy. Autophagy has also recently been shown to be involved in diverse aspects of the innate and adaptative immune response (reviewed in [2]).

### 1.1. Autophagosome Formation and Expansion

**Figure 1.**
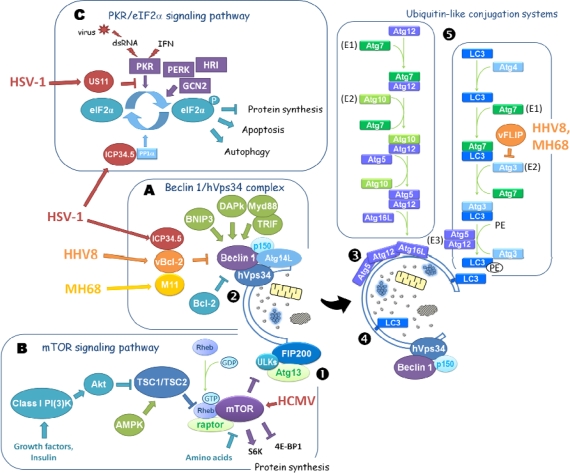
The autophagic machinery and its regulation pathways. Autophagosome formation is controlled by the ULKs-FIP200-Atg13 complex (1) and the Beclin 1:hVps34:Atg14L complex (2). Expansion and closure of the autophagosomal membrane are dependent on the Atg5-Atg12-Atg16L complex (3) and on the mammalian homolog of Atg8, LC3 (4). Two ubiquitin-like conjugation systems (5) lead to the conjugation of Atg12 to Atg5, and of LC3 to phosphatidylethanolamine (PE). The Atg5-Atg12 conjugate associated with Atg16 contributes to the stimulation of LC3 conjugation. Some important signaling pathways involved in the regulation of autophagy are presented here. (A) Beclin 1 is a platform protein that can interact with a variety of cellular proteins. Positive regulators of Beclin 1 are boxed in green. The interaction of Beclin 1 with anti-apoptotic proteins of the Bcl-2 family blocks the induction of autophagy. (B) In the presence of amino acids, insulin, growth factors and energy, mTOR represses autophagy by inhibiting the kinase activity of ULKs. In contrast, in the absence of amino acids, growth factors and/or activation of the AMPK, mTOR is inhibited and autophagy initiated by the ULKs-FIP200-Atg13 complex. (C) The kinase PKR, activated by viral infections via double stranded RNA (dsRNA) and IFN, leads to the phosphorylation of eIF2α. Phosphorylated eIF2α allows host shutoff of the protein synthesis, and the induction of apoptosis and stimulation of autophagy to occur. Some autophagy proteins are targeted by Herpesvirus-encoded proteins. ICP34.5 (HSV-1), vBcl-2 (HHV8) and M11 (MH68) inhibits autophagy by interacting with Beclin 1. In order to block autophagy, ICP34.5 is also able to recruit the phosphatase 1 alpha PP1α, leading to the dephosphorylation of eIF2α. Us11 (HSV-1) blocks PKR directly in the late stages of infection. vFLIP (HHV8, MH68) inhibits autophagy by interacting with Atg3. HCMV may inhibit autophagy *via* mTOR activation.

During autophagy, part of the cytoplasm is engulfed by a double membrane (the isolation membrane), which first elongates and then closes to form the autophagosome (Figure 1). Autophagosomes use dynein motors to travel along the microtubule network towards the microtubule-organizing center, where they merge with endosomes (to form amphisomes) or with lysosomes (to form autolysosomes). Next, the inner autophagosomal membrane and the sequestered material are degraded by lysosomal hydrolases. The origin of the autophagosomal membrane is still uncertain, but there is growing evidence that the Endoplasmic Reticulum (ER) provides lipids for growing autophagosomes [3,4]. So far, more than 30 AuTophaGy-related (Atg) genes have been identified in yeast, and most of them are conserved in eukaryotes [5]. The formation of autophagosomes involves 18 different Atg genes.

The nucleation and assembly of the initial phagophore membrane is a highly regulated process mediated by two complexes. (i) A stable complex formed by ULK1/2, Atg13, FIP200 and a novel Atg13-binding protein, known as Atg101, which localizes to the phagophore during starvation in mammalian cells [6,7]. This complex mediates mTOR (mammalian Target of Rapamycin) kinase-signaling to the autophagic machinery [8,9]. Its counterpart in yeast, the Atg1-Atg13-Atg17 complex, is also essential for the regulation of autophagy, and integrates regulation from TOR and diverse signaling pathways [10–12]. (ii) The Beclin 1:hVps34 macromolecular complex is required for the nucleation, and assembly of the initial phagophore membrane (Figure 1). This complex consists of the class III phosphatidylinositol 3-kinase (PI3K) (or hVps34), its regulatory protein kinase p150 or hVps15, Beclin 1 (Atg6 in yeast), and Atg14L (or Barkor and Atg14 in yeast) [13,14].

This complex, and other Atg proteins, recruit the Atg5-Atg12-Atg16L multimeric complex, and the lipidated form of LC3 (microtubule-associated protein light chain 3), known as Atg8 in yeast, to the phagophore, resulting in membrane elongation. These last two components result from two sequentially acting ubiquitin-like conjugation systems (Figure 1). In the first conjugation system, Atg12 is activated by the E1-like enzyme, Atg7, and conjugated to Atg5 by the E2-like enzyme, Atg10. Atg12-Atg5 binds non-covalently to Atg16L, to form the Atg16L-Atg12-Atg5 complex which associates with the membrane [15–18].

The second conjugation system involves the Atg7 (E1-like) and Atg3 (E2-like) proteins that mediate the conjugation of LC3 (previously processed by Atg4) to phosphatidylethanolamine (PE) [19]. Opposite to the unconjugated cytosolic LC3 form I, the lipidated form of LC3 (also known as LC3 form II) is associated with the autophagosomal membranes [20]. The conjugation of LC3 (detected by Western blot analysis), and the membrane localization of lipidated LC3 (detected by fluorescent microscopy) are the usual markers of early autophagy [21]. Readers interested in methods used for monitoring autophagy can consult a comprehensive review on this topic [21].

It has been shown recently that the Beclin 1:hVps34:p150 core complex is in fact involved in the various stages of autophagy, and the specificity of the complex is conferred by binding to Atg14L, to UVRAG, or to UVRAG and Rubicon [13]. Atg14L recruits the complex to the autophagosomal membrane, and is necessary for autophagosome formation, whereas the Beclin 1:hVps34:p150 complex formed with UVRAG is involved in autophagosome and endosome maturation. In contrast, when UVRAG is combined with Rubicon, it prevents the maturation of autophagosomes and endosomes [22].

### 1.2. Regulation of Autophagy

The mTOR kinase is essential for the control of autophagy by growth factors, insulin, nutrients, calcium signaling, ATP level, hypoxia and oxidative stress [23]. This key inhibitor of autophagy is present in two different complexes: mTORC1 and mTORC2, where it is associated with raptor and rictor, respectively. The mTORC1 complex controls protein synthesis, the importation of nutrients and autophagy. Inhibition of mTORC1 by rapamycin activates autophagy in all eukaryotic cells. The downstream targets of mTORC1 that regulate autophagy in mammalian cells are ULK1 and ULK2 [24]. Upstream, mTORC1 is itself inhibited by the heterodimer TSC1/TSC2 (TSC: tuberous sclerosis complex). TSC2 is a GTPase-activating protease acting on Rheb, which in its inactive GDP-form disassembles from mTORC1, resulting in its inactivation [25]. The mTORC1 complex integrates signals from multiple upstream signaling pathways. Growth factor signaling occurs via class I PI3K, and Akt/PKB (serine/threonine protein kinase B). Phosphorylation of TSC2 by Akt/PKB inactivates the TSC1/TSC2 complex, which in turn activates mTORC1. The mTORC1 complex is also activated by high amino acid levels via the stimulation of the heterodimer of Ras-related small GTPases (RAG). In contrast, activation of the energy sensor AMPK by an increase in the AMP/ATP ratio inactivates mTORC1 by activating TSC2. The second complex of mTOR, mTORC2, has been reported to regulate autophagy via the transcription factor FoxO, in the context of muscle atrophy [26,27].

The Beclin 1:hVps34 complex is also modulated by the recruitment of positive and negative regulators, by post-translational modifications of Beclin 1 [28], and by microRNA as recently reported [29]. In the last few years, we have seen a huge increase in the number of Beclin 1 interaction-partners identified. Anti-apoptotic factors of the Bcl-2 protein family (Bcl-2, Bcl-xl) are the most abundantly documented repressors of Beclin 1. Under normal conditions, Bcl-2 interacts with Beclin 1, thus inhibiting autophagy [30]. In response to amino acid deprivation or ceramide treatment, the phosphorylation of Bcl-2 by JNK1 leads to reduced interaction between Bcl-2 and Beclin 1, which in turn triggers autophagy [31,32].

Similarly, several factors such as DAP kinase, TRIF or MyD88 (myeloid differentiation factor 88), and BNIP3, which are activated by death signals, pathogens and hypoxia, respectively, enhance autophagy by reducing/disrupting the Beclin 1/Bcl-2 interaction [28,33,34]. MyD88 and TRIF are adaptor proteins recruited by activated toll-like receptors (TLRs) [34], and they are involved in the stimulation of autophagy induced by pathogens via TLRs [35]. Several viral proteins, such as ICP34.5 (HSV-1) or vBcl-2 (HHV-8), have been shown to interact with Beclin 1, leading to the inhibition of autophagy, and will be discussed later in this review [36].

Autophagy is also upregulated by the stress-induced eIF2α kinase signaling pathway [37,38]. However, the way the eIF2α kinase signaling impacts on the autophagic machinery is still unknown. The eIF2α kinases are evolutionarily-conserved serine/threonine kinases that inhibit translation by phosphorylating and inhibiting the eukaryotic initiation factor 2 alpha (eIF2α). In mammalian cells, four eIF2α kinases, GCN2, PKR, PERK and HR1, activated by amino acid starvation, viral infection, ER stress and heme deprivation, respectively, are described [37]. It has been proposed that phosphorylation of eIF2α by GCN2 may trigger autophagy in yeast and in fibroblasts [38]. Double-stranded RNA-activated protein kinase (PKR) is inducible by interferon (IFN), and can be activated by double-stranded RNA, a common intermediate in the replication of many viruses. PKR is a key player in the antiviral action of interferon, and many viruses express proteins that antagonize the PKR signaling pathway [39]. Activation of the PKR kinase has been shown to be involved in autophagy induction in murine fibroblasts infected with an HSV-1 mutant virus [38].

Recent data suggest that activation of eIF2α kinase by PERK after the expression of misfolded polyglutamine 72 repeat (polyQ72) induces autophagy and upregulates Atg12 mRNA [40]. Other studies have also reported that autophagy is induced by ER stress but, in contrast, that autophagy is activated via the IRE-1-JNK pathway and not via PERK in neuroblastoma cells treated with ER stressors [41]. The signaling pathways that link ER stress to autophagy might therefore be cell-dependent.

### 1.3. Autophagy and Viruses

Several recent reviews address the interplay between viruses and autophagy [39,42,43]. Autophagy protects host cells against viral attack by degrading the viruses in autolysosomes, or by activating the innate immunity of the cells by loading viral components onto endosomal sensors such as TLRs [35,44]. It has been suggested that autophagy may have a deleterious effect on viral pathogenesis in the case of the neurotropic Sindbis virus [45], and the role of autophagy has been clearly demonstrated to limit the replication of the tobacco mosaic virus [46]. Autophagy is also involved in the adaptative immune responses to microorganisms infection, for example by providing viral endogenous antigens for loading onto major histocompatibility complex (MHC) class I and class II in order to activate the adaptive immunity [47–49]. Some viruses have been shown to have developed diverse tactics to antagonize the host cell’s autophagic defense. Most of them belong to the *Herpesviridae* family, and the mechanisms they employ are discussed later in this article. Inhibition of autophagy has also been reported in HIV-infected CD4 T-lymphocytes [50]. Moreover, HIV has been shown to inhibit the fusion of autophagosomes with lysosomes in macrophages through interaction of the viral protein Nef with Beclin 1, leading to an accumulation of autophagosomes in the cytoplasm [50,51].

In some situations autophagy is also able to act as a pro-viral pathway that helps viruses to replicate or exit from cells [52]. For example, stimulation of autophagy has been reported to increase the yields of poliovirus, hepatitis C virus (HCV), Dengue virus and Coxsackie B virus [53–55]. It has also been suggested that autophagy may help poliovirus particles to exit from the cell during the late stages of infection [56]. HCV induces the accumulation of autophagosomes in infected cells, but inhibits their fusion with lysosomes [54], and seems to require autophagy at an early stage of the infection [55].

## 2. Autophagy is Modulated by Herpesviruses

Autophagy is activated by Herpesviruses, and thus may play an important role in the antiviral cell defense either by clearing intracellular viruses (xenophagy) or by activating antigen presentation [42]. It is not therefore surprising that Herpesviruses have evolved mechanisms to antagonize autophagy. Some of them have developed proteins that inhibit PKR-eIF2α signaling, which triggers autophagy as part of a global antiviral IFN response, but an increasing number of Herpes viral proteins have been reported to target the autophagic machinery more specifically. However, a few cases have also been documented, in which Herpesviruses are not able to inhibit autophagy [47,57].

### 2.1. Alphaherpesvirinae

#### 2.1.1. Herpes Simplex Virus Type 1 (HSV-1)

The regulation of autophagy by HSV-1 infection was first reported in 2002 [38], and has now been well documented, using both *in vitro* and *in vivo* approaches. HSV-1 is known to be able to antagonize the host autophagy response in fibroblasts [38], in primary murine neurons [44], and in macrophages [47]. However, during the late phase of macrophage infection, HSV-1 stimulates autophagosome formation. Taken together these data suggest that the modulation of autophagy by HSV-1 could be either cell-type dependent and/or time dependent. In fibroblasts and neurons, HSV-1 counteracts the induction of autophagy via Infected Cell Protein 34.5 (ICP34.5), which is encoded by the neurovirulence gene gamma_1_34.5, and which acts by two separate mechanisms: binding to Beclin 1, and antagonizing autophagy-stimulating PKR signaling [36]. The HSV-1 protein ICP34.5 has been shown to block the translational repression of PKR by recruiting a cellular phosphatase, PP1α, which leads to PKR-mediated eIF-2α dephosphorylation despite the presence of activated PKR [58]. A deletion mutant HSV-1 virus, which lacks the γ_1_34.5 gene, strongly activates PKR signaling, and causes the premature shutoff of protein synthesis in various different cell types, and is neuroattenuated in mice [59]. The first report of an impact of ICP34.5 on autophagy was that of Tallóczy *et al*., who discovered that the PKR-eIF-2α pathway regulates positively autophagy [38]. Indeed, an ICP34.5 deletion mutant virus has been shown to promote autophagy in murine embryonic fibroblasts (MEFs), unlike the wild type virus, but it no longer triggers autophagy in the PKR*−/−* and Ser-51 nonphosphorylatable mutant eIF2α MEFs [38]. Taken together, these data show that HSV-1 infection activates PKR and stimulates autophagy, but that the expression of ICP34.5 allows the virus to block PKR signaling and so to antagonize the induction of autophagy (Figure 1). Interestingly, the autophagic protein Beclin 1, which was initially identified as a Bcl-2 interacting protein (35), has also been reported to interact with ICP34.5 in a yeast two-hybrid screen using ICP34.5 as bait (B. Roizman, personal communication). Following on from this, Levine and colleagues reported recently that ICP34.5 binds directly to human Beclin 1, and inhibits its autophagy function (Figure 1) [36]. In the absence of HSV-1 infection, the expression of ICP34.5 is sufficient in itself to block starvation-induced autophagy in both yeast and mammalian cells. The ICP34.5 binding-domain of Beclin 1 has not yet been clearly identified, but is known to differ from the Bcl-2-binding domain. In contrast, the Beclin-1 binding-domain of ICP34.5 maps to amino-acids 68–87, and is distinct from the GADD34-related region involved in the dephosphorylation of eIF2α.

##### 2.1.1.1. Impact of Autophagy on HSV-1 Viral Replication

It is tempting to speculate that autophagy is inimical for virus life, since HSV-1 has developed various strategies to block this cellular process. However, the impact of autophagy on HSV-1 replication is still controversial. Talloczy *et al*. reported that the growth of an ICP34.5 deletion mutant virus was defective in normal cells, whereas in PKR*−/−* MEFs, replication of this mutant virus could be restored [44]. In PKR−/− cells, autophagy is functional but cannot be induced by viral infection. This had already been reported previously, but had been attributed to the role of ICP34.5 in inhibiting PKR-dependent host cell shutoff [60]. However, in autophagy-deficient Atg5−/− MEFs, deficient growth of a ICP34.5 deletion mutant virus was not abolished [61]. These latest observations tend to suggest that autophagy does not in fact have any impact on the viral replication cycle in cell cultures, and so cannot be responsible for the growth defect of the ICP34.5 deletion mutant virus.

However, *in vivo* investigations of the role of ICP34.5 have indicated that autophagy might contribute to the pathogenesis of HSV-1 [36]. ICP34.5 was initially identified in Roizman’s laboratories as the neurovirulence factor of HSV-1, [59]. It has been proposed that ICP34.5 contributes to neurovirulence by blocking the host cell shutoff mediated by PKR eIF2α signaling [62]. Supporting this surmise, an ICP34.5 deletion mutant virus displayed wild-type virulence in mice lacking PKR [63]. However, a Beclin 1-binding-deficient ICP34.5 mutant virus is highly neuroattenuated in mice [36]. These findings show that the ICP34.5/Beclin 1 interaction, and consequently the inhibition of autophagy by ICP34.5 might be another factor that contributes to viral neurovirulence, independently of the role of ICP34.5 in antagonizing host cell shutoff. Furthermore, the neurovirulence of Beclin 1-binding-deficient ICP34.5 HSV-1 was fully restored in PKR−/− mice, suggesting that PKR is connected to Beclin 1 in the regulation of autophagy. Further studies will be necessary to elucidate the complex relationships between neurovirulence, ICP34.5 and autophagy control.

Interestingly, the HSV-1 Us11 gene is also dedicated to regulating the PKR eIF2α signaling pathway [64,65]. The Us11 protein is a double-stranded RNA binding protein, expressed with late kinetics, which can antagonize PKR either by a direct interaction or by interacting with PACT, an activator of PKR [64,66]. Immediate early kinetic expression of the Us11 protein in an ICP34.5 deletion mutant virus restores the ability of the virus to reverse eIF2α phosphorylation and the shutoff of protein synthesis [67]. This mutant virus, which does not express ICP34.5, is able to inhibit virus-induced and PKR-mediated autophagy (our unpublished results).

###### 2.1.1.2. Antiviral Mechanisms of Autophagy

The way autophagy contributes to the antiviral innate immune response in the context of HSV-1 infections is still puzzling. Talloczy *et al*. reported that xenophagy plays a role in degrading HSV-1 virions, based on the presence of virions visualized by electron microscopy inside autophagosomes, and on the accelerated degradation rate of viral proteins after infection with an ICP34.5 deletion mutant [44]. However, these ultrastructural studies were done at a late stage in the infection, and the entrapped virions corresponded to *de novo* synthesized viral particles. Degradation of both structural and non structural viral proteins is enhanced in the context of unrestrained autophagy, but it is not necessarily correlated with the degradation of entire HSV-1 particles [44]. The ability of xenophagy to capture and eliminate new incoming HSV-1 virions therefore remains to be demonstrated.

**Figure 2.**
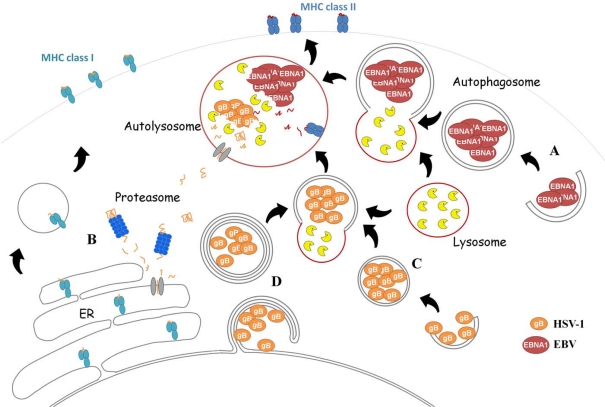
Contribution of autophagy to adaptative immunity against Herpesviruses. There are two examples of autophagy-dependent MHC presentation of herpesvirus proteins [47,48]. The EBV protein EBNA1 is presented onto MHC class II molecules via autophagy (A). In HSV-1 infected macrophages, the glycoprotein gB is processed both through the classical MHC class I presentation pathway involving the proteasome (B) and through a vacuolar pathway involving autophagy and partially lysosomal digestion. The glycoprotein gB was detected in classical autophagosomes (C) as well as in newly discovered four-layered membrane autophagosomes derived from the nucleus (D).

Autophagy might rather play a key role in the adaptive immune response by generating peptides from trapped virions for presentation by the MHC class I and II molecules [47,68]. Indeed, a recent paper has reported that the neurovirulence of the Beclin 1-binding-deficient ICP34.5 mutant virus was completely restored in mice lacking functional T and B cells, clearly connecting autophagy to the control of adaptative immune response [68]. English *et al*. have also reported that autophagy is involved in the presentation of the viral glycoprotein gB to CD8+ T cells by MHC class I, in collaboration with the canonical ER-dependent pathway [47]. These authors have also shown that the infection of macrophages by HSV-1 leads to an ICP34.5 dependent inhibition of autophagy in the early infection phase, followed by stimulation of the autophagic pathway in the late phase. Interestingly, these late phase autophagosomes consisted of both classical autophagosomes and four-layered membrane structures emerging from both the inner and outer nuclear membranes. Their findings showed that the processing of glycoprotein gB for MHC class I presentation in HSV-1 infected macrophages occurs in two phases: first via the classical pathway involving proteasome-mediated degradation during the early infection phase, and then via this newly-characterized nuclear membrane-derived autophagy during the late infection phase (Figure 2) [69].

##### 2.1.2. Bovine Herpes Simplex Virus Type 1 (BHV-1)

Recent findings suggest that BHV-1 may have developed a strategy to evade autophagy, as has its cousin, HSV-1. Kidney cells infected by a mutant BHV-1 virus lacking bovine Infected Cell Protein 0 (bICP0) displayed more autophagosomes than cells infected with the wild-type virus, indicating that bICP0 might antagonize autophagy [70]. This bovine homolog of ICP0 has also been reported to protect kidney cells against UV-induced apoptosis, since a bICP0 null mutant virus was unable to inhibit UV-induced apoptosis [70].

##### 2.1.3. Varicella-zoster Virus (VZV)

A recent paper reports that VZV stimulates autophagy during the late stages of infection in both fibroblasts and melanoma cells [57]. Interestingly this stimulation of autophagy has also been corroborated by *in vivo* data, since LC3 was expressed abundantly in infected cells taken from biopsies of zoster vesicles. Therefore, in contrast to HSV-1, VZV appears to be unable to evade the host’s autophagy machinery. Moreover, no ortholog of the HSV-1 γ_1_34.5 gene, or viral Bcl-2 homolog, such as those known to be present in gammaherpesviruses, could be detected in the VZV genome. Although the physiological relevance of VZV-induced autophagy is still unknown, Takahashi *et al*. suggest that autophagy could be responsible for the low yields of cell-free VZV [57]. VZV is in fact known to remain highly cell-associated in cell cultures, and enveloped virions accumulate in acidic cytoplasmic vacuoles other than lysosomes, which means that autophagy might limit viral production by trapping viral particles in the autophagosomal pathway.

#### 2.2. Betaherpesvirinae

##### 2.2.1. Human Cytomegalovirus (HCMV)

HCMV is a widespread opportunistic pathogen that causes severe diseases and death in newborn infants and immunocompromised individuals. By monitoring autophagosome formation and autophagic flux, a recent study has reported that HCMV is able to counteract the autophagic antiviral response in human embryonic fibroblasts after 24 hours of infection, dependently of viral protein synthesis [71]. Despite a dramatic decrease in the number of autophagosomes after HCMV infection, the expression and conjugation of LC3 are both increased, suggesting that HCMV achieves the fine modulation of autophagy by inhibiting some specific steps in the autophagic pathway and enhancing others [71]. The precise mechanisms involved in the inhibition of autophagy by HCMV are still unclear. However, it has been reported that HCMV activates autophagy-inhibiting mTOR signaling [71,72], and inhibits autophagy-stimulating PKR signaling via the interaction between two related viral proteins, TRS1 and IRS1, with PKR and dsRNA [73]. These signaling pathways could therefore be involved in the inhibition of autophagy. The fact that HCMV-infected cells are resistant to autophagy induced by rapamycin, an mTOR inhibitor, and by lithium chloride, which acts independently of mTOR, also suggests that HCMV targets several regulatory pathways [71]. In view of the interplay known to exist between apoptosis and autophagy [74,75], apoptosis-inhibitory HCMV gene products, such as vMIA, VICA or U_L_38, could also be involved in the control of autophagy by HCMV [76]. Notably, the product of the early gene U_L_38 might prevent autophagy via mTOR signaling, since pU_L_38 has been shown to bind TSC2 and to antagonize the ability of the TSC1/TSC2 complex to downregulate mTOR complex 1 [77]. The inhibition of autophagy by HCMV appears to be very complex since it involves several regulation pathways and is time dependent. Indeed, autophagy is induced during the first hours of infection, independently of the expression of viral proteins, because this stimulation is observed even in the context of cells infected with UV-inactivated HCMV (our unpublished data).

#### 2.2.2. Human Herpesvirus (HHV-6)

HHV-6, which belongs to the *Roseolovirus* genus of the β–Herpesvirus subfamily, is related to HHV-7 and, to a lesser extent, to HCMV. The impact of HHV-6-infection on autophagy is still unexplored. It is noteworthy that HHV-6 uses human CD46 as a cellular receptor during fusion with and entry into target cells [78]. CD46, which is well known to act as a receptor for several pathogens, has been reported very recently to induce autophagy via a pathway involving the Beclin 1 complex after measles virus infection [79]. These findings suggest that HHV-6 infection could also modify the level of autophagy.

#### 2.3. Gammaherpesvirinae

##### 2.3.1. Epstein-Barr Virus (EBV)

EBV infects the oral epithelium, where it undergoes lytic replication, and resting B lymphocytes, where it establishes long-term latency. Up to nine EBV proteins are expressed during latency: six EBV-encoded nuclear antigens (EBNA-1-6), and three latent membrane proteins (LMP-1, -2A, -2B). Expression of these latent proteins contributes to EBV properties for proliferation and/or transformation of host cells, predisposing them to numerous malignancies. Nothing is yet known about the interplay between autophagy and the lytic cycle of EBV, but at least two latent antigens, EBNA 1 and LMP1, are known to activate or to interfere with the autophagic machinery [48,80].

###### 2.3.1.1. EBNA1 Is Loaded onto MHC Class II after Macroautophagy

EBNA1 is essential for latency, since it enables the replication and persistence of EBV genomes in latently infected cells, and activates the expression of other latency genes. Being an endogenous antigen, EBNA1 would be expected to be loaded onto MHC class I after proteasomal processing. However, in fact EBNA1 inhibits its own proteasomal-mediated turnover, and consequently its presentation on MHC class I [81]. In contrast, EBNA1 elicits a CD4+ T cell immune response after intracellular MHC class II processing. Paludan *et al*. have demonstrated that autophagy contributes to MHC class II-EBNA1 presentation in EBV-transformed lymphoblastoid cells [48] by delivering this EBV antigen to the lysosomal compartment, where the processing occurs. Inhibition of lysosomal acidification induces an accumulation of EBNA1 in autophagosomes, as revealed by ultrastructural analysis of EBNA1-transfected Hodgkin’s lymphoma cells, while suppression of autophagy by 3-methyladenine or by small interfering RNA restrained the intracellular processing of EBNA1, and consequently its MHC class II presentation. In contrast, the MHC class II presentation of two other EBV latent proteins, EBNA2 and EBNA3C, does not involve autophagy, but depends on intercellular antigen transfer [82]. Endogenous MHC class II processing after autophagy has also been reported for other proteins, complement C5, Mucin 1 oncoprotein and a bacterial antigen neomycin phosphotransferase [83]. However to date, EBNA1 is the only viral antigen known to be presented on MHC class II via the autophagic pathway.

###### 2.3.1.2. Latency-associated LMP1 Induces Autophagy

LMP1 is a viral protein of EBV expressed in latency, and in many EBV-associated tumors, including non-Hodgkin lymphoma. This oncogene has been shown to drive proliferation of EBV-infected B cells via a signaling pathway similar to that of CD40 [84]. LMP1 is expressed at a wide range of levels in B-cells originating from the same clone, and Lee and Sugden showed that this variation controls the induction of different signaling pathways: intermediate levels of LMP1 expression are required for B-cell proliferation to occur, whereas higher levels induce the unfolded protein response (UPR), by activating the PKR-like endoplasmic reticulum-related kinase (PERK) [85]. UPR activation is thought, in turn, to drive the synthesis of LMP1 [85]. The same authors reported that the expression of LMP1 induced autophagy in both EBV-infected B cells and EBV-negative cells expressing exogenous LMP1 [80]. The stimulation of autophagy was also correlated with the level of LMP1 expression: cells expressing low levels of LMP1 displayed autophagosomes, while those expressing higher levels displayed autolysosomes. The transmembrane domains 3–6 of LMP1 were shown to be responsible both for inducing autophagy and activating PERK. The UPR, which is induced by the accumulation of unfolded proteins in the ER, includes the activation of PERK and transcription factor 6 (ATF6), and the induction of inositol requiring kinase 1 (IRE-1). Interestingly, several studies have reported the induction of autophagy by ER stress, notably after activation of eIF2α by PERK, thus linking autophagy to the UPR [37,40]. The signaling pathway leading from LMP1 to autophagy is still unclear, but might involve PERK activation by the transmembrane domain of LMP1. Further investigations are necessary to clarify the exact roles of autophagy in EBV-infected cells. Lee and Sugden have proposed that autophagy might regulate the turnover of LMP1 by delivering it to lysosomes [80]. Indeed, inhibiting autophagy promoted the accumulation of LMP1, and induced a slight decrease in cell proliferation. Therefore, the activation of autophagy and UPR by EBV both seem to be responsible for the variable LMP1 levels.

##### 2.3.2. Viral Bcl-2 Homologs of γHerpesviruses Are both Antiapoptotic and Antiautophagic

Apoptosis is used by host cells as part of their antiviral response, and impairs virus production by triggering premature cell death. As a consequence, many viruses have developed strategies to counteract virus-induced apoptosis. *Gammaherpesvirinae*, for example, encode viral homologs of the cellular antiapoptotic proteins Bcl-2 and FLIP (FLICE-like inhibitor protein) to prevent host cell death. However, recent findings have demonstrated that these proteins also suppress autophagy [30,86–88]. Although autophagy is a recognized cell survival mechanism, there is also evidence of an interconnection between autophagy and apoptosis [75,89] and, for example, several mediators of apoptosis have been shown to control autophagy. Therefore, it is not unexpected to find that some antiapoptotic proteins also repress autophagy. Viral Bcl-2 homologs, vBcl-2, M11 and BHRF1, have been reported in HHV-8, mouse Herpesvirus strain 68 (HV-68) and EBV respectively [90–92]. Several results indicate that these proteins are essential for γ-Herpesvirus pathogenesis, but so far M11 is the only γ-herpesvirus Bcl-2 homolog that has definitely been shown to play a role during infection *in vivo* [93]. Pattingre *et al*. were the first to demonstrate that cellular and HHV-8 Bcl-2s inhibit autophagy through interacting with the BH3 domain of Beclin 1 [30]. Subsequently, the Bcl-2 homolog of γHV68 was also reported to inhibit autophagy by interaction with Beclin 1 [86,87]. The antiautophagic properties of the EBV Bcl-2 homolog, BHFR1, have not yet been investigated. The autophagy-inhibitory-complex formed by cellular Bcl-2 and Beclin-1 can be modulated by nutrient conditions, whereas viral Bcl-2 forms a stable association with Beclin-1, which results in a constant repression of autophagy [31]. Starvation leads to dissociation of the cellular Bcl-2-Beclin 1 complex as a result of the phosphorylation of cBcl-2 by activated JNK1, and consequently an induction of autophagy [31]. JNK1 phosphorylates cellular Bcl-2 at three sites present in the nonstructured loop, which are absent in the viral Bcl-2 proteins. Moreover, M11 has been reported to bind to Beclin 1 more strongly than cellular or HHV-8 Bcl-2 [87]. The enhanced anti-autophagic activity of viral Bcl-2 suggests that autophagy plays an important role as an antiviral cellular defense mechanism. It has also been proposed that viral Bcl-2 proteins may inhibit Beclin 1-mediated tumor suppression, and so the inhibition of autophagy might promote oncogenic events [89].

The regulatory protein FLIP, which contains death effector domains (DED) such as the adaptator protein FADD and the procaspases 8 and 10, regulates apoptosis from death receptors [94]. Cellular FLIP (cFLIP), and viral FLIP (vFLIP) of HHV-8 and Herpesvirus Saimiri, have recently been shown to inhibit autophagy induced by starvation or rapamycin, similarly to Bcl-2 [88]. FLIP interacts with the E2-like enzyme Atg3 and represses autophagy by preventing the binding of Atg3 to LC3. Consequently, FLIP prevents the conjugation of LC3 which is essential for autophagic vesicle expansion. Interestingly, rapamycin, a known inducer of autophagy, displays tumor suppressor activity against HHV-8 induced Kaposi’s sarcoma and primary effusion lymphoma. FLIP expression prevented autophagic cell death induced by rapamycin, hinting that autophagy inhibition may be relevant for *in vivo* HHV-8 pathogenesis [88]. Thus, γ–Herpesviruses, via interaction of vBcl-2 and vFLIP with Beclin 1 and Atg3 respectively, impact two stages of autophagosome formation [88]. The fact that the γ–herpesviruses have evolved proteins that inhibit autophagy at various different steps highlights the importance of controlling this mechanism. The significance of this inhibition is not yet known for the pathogenesis of the *Gammaherpesvirinae* subfamily, but might be linked to the oncogenic potential of these viruses, and autophagy and apoptosis might execute protective functions in concert.

## 3. Conclusions

All three subfamilies of *Herpesviridae* have evolved distinct strategies to block the induction of autophagy in infected cells (table 1). Evasion of host autophagy occurs by direct inhibition of the autophagic machinery via interaction with Beclin 1 (HSV-1 ICP34.5 or the viral Bcl-2 homologs of γ–herpesvirus), with Atg3 (γ–herpesvirus FLIP homolog) or via regulation pathways including activation of the inhibitory mTOR pathway (HCMV) and inhibition of the PKR/eIF2α pathway (HSV-1). The same host autophagy protein, Beclin 1, is targeted by different herpesviruses and via diverse strategies, which is not surprising given the central role of Beclin 1 in the regulation of autophagy. More remarkably, the same viral protein (HSV-1 ICP34.5) prevents the induction of autophagy by two different routes. The resourcefulness shown in strategies that target autophagy underscores the importance for Herpesviruses of blocking this cellular mechanism.

In most cases, Herpesviruses seem to be able to overcome cellular autophagy, but few examples have also been documented of Herpesviruses that are not able to control autophagy [47,57]. VZV actually stimulates autophagy during late stages of infection, but the fact that VZV does not encode an anti-autophagic protein could account for this [57]. Autophagy induction has also been reported in macrophages infected with HSV-1, whereas this same virus is able to block autophagy in neurons and in fibroblasts [36,47].

Autophagy has been reported to produce some antiviral effects against Herpesviruses, including xenophagy, *i.e.,* the direct degradation of viruses in autolysosomes [44], and viral antigen presentation on MHC class I and II [47,48], but other mechanisms could also be involved. While the deleterious effect of autophagy on *in vitro* viral growth is not obvious [61], some studies do seem to indicate that autophagy helps to restrict viral pathogenesis, such as the neurovirulence of HSV-1 [36] or the virus-induced oncogenesis of certain gamma Herpesviruses [89]. Autophagy has recently also been demonstrated to impede *in vivo* viral growth by activating the adaptive immunity [68].

Herpesviruses are major pathogens which are able to persist throughout the lifetime of the infected host by establishing latent infections. No currently available drugs eradicate these viruses, since anti-herpes drugs target viral genome replication. It is crucial to find out how autophagy acts as an antiviral defense mechanism against herpesviruses, because this mechanism could provide a target for a new therapeutic approach. Interestingly, rapamycin, which is known to induce autophagy, has been reported to decrease the incidence and gravity of HCMV infections after organ transplantation [95,96]. Similarly, rapamycin treatment seems also detrimental to the pathogenesis of gamma Herpesviruses, such as EBV and HHV-8. However, because rapamycin also targets pathways other than autophagy, it is difficult to isolate the role of autophagy stimulation in its therapeutic action. New agents that specifically target autophagy will have to be developed to test this potential new antiviral approach. Future studies will also have to find out more precisely when and how autophagy prevents Herpesvirus pathogenesis.

## Figures and Tables

**Table 1.** Relationships between Herpesvirus and autophagy.

**Herpesvirus**	**Viral evasion of autophagy**	**Autophagy promotion**	**Host-virus interactions and host antiviral defense**	**Viral proteins/mechanisms**	**Ref.**
**Alphaherpesvirinae**					

**Herpes simplex virus type 1 (HSV-1)**	HSV-1 infection inhibits autophagy in neurons and in fibroblasts	HSV-1 infection induces autophagy in macrophages	Viral control of autophagy is involved in neurovirulence	ICP34.5 blocks autophagy by inhibiting PKR signaling and by direct interaction with Beclin 1	[38] [36] [61]
			Autophagy contributes to MHC class I presentation of gB to CD8+ cells	gB	[47]

**Bovine herpesvirus type 1 (BHV-1)**	BHV-1 WT may inhibit autophagy whereas the bICP0 null mutant may induce autophagy in MDBK cells			bICP0 may block autophagy	[70]

**Varicella-zoster Virus (VZV)**		VZV stimulates autophagy in fibroblasts and *in vivo* in human zoster vesicles		Unknown	[57]

**Betaherpesvirinae**					

**Human cytomegalovirus (HCMV)**	HCMV inhibits autophagy in human fibroblasts			May activate mTOR pathway?	[71]

**Gammaherpesvirinae**					

**Epstein-Barr virus (EBV)**		A latency-associated protein (LMP1) induces autophagy	Autophagy contributes to MHC class II presentation of EBNA1 to CD4+ cells	LMP1 Proposed role in regulating its own expression level	[80]
				Latency associated protein EBNA1	[48]

**Human Herpesvirus 8 (HHV8), murine □-HV68**	HHV8 may inhibit autophagy		Unknown	Viral homologs of Bcl-2 (vBcl-2, M11) inhibit autophagy by its interaction with Beclin 1	[30] [86] [89]
				Viral homologs of FLIP (vFLIP) inhibit autophagy by interaction with Atg3	[88]
